# Lithium-induced neurotoxicity (SILENT Syndrome) in a Schizoaffective patient: A case of prolonged treatment leading to severe neurological complications

**DOI:** 10.1192/j.eurpsy.2025.1731

**Published:** 2025-08-26

**Authors:** E. García, J. Tortajada, J. Santilari

**Affiliations:** 1 Hospital Universitari Institut Pere Mata de Reus; 2 CIBERSAM; 3 Institut d’Investigació Sanitària Pere Virgili, Reus, Spain

## Abstract

**Introduction:**

A 71-year-old female patient with schizoaffective disorder, under psychiatric follow-up and treated with lithium for over 20 years (with successive stable therapeutic blood levels), presented to the emergency department with symptoms of temporospatial disorientation, aphasia, gait disturbances, tremor, and fluctuating encephalopathy. Symptoms have progressively worsened since she was found on the floor of her home one month ago. Subsequent complementary examinations (clinical evaluation, cranial CT scan, and laboratory tests) returned normal results.

Given the clinical picture presented, a differential diagnosis was made including neurocognitive, toxic-metabolic, psychiatric, and pharmacological causes (lithium neurotoxicity). Initially, the treatment was adjusted by reducing the lithium dose (from 600mg to 400 mg/day) and the associated medication was modified (mirtazapine and lormetazepam). Additional investigations or hospital admission were recommended to rule out an underlying pathology.

**Objectives:**

The aim of this study is to explore and discuss the differential diagnosis and symptoms associated with lithium neurotoxicity.

**Methods:**

We conducted a literature review through the PubMed database.

**Results:**

The patient’s symptoms worsened, and she was admitted to a long-term care facility. Active psychiatric pathology and cognitive decline were ruled out; renal function, thyroid function, and cranial MRI were normal. However, neurological symptoms and disorientation persisted.

The lithium dose was reduced to 200 mg/day, which led to the onset of manic symptoms. Upon increasing the dose back to 400 mg/day, the patient exhibited decreased consciousness, and serum lithium levels increased to 1.24 mEq/L, despite previously stable levels at this dose. Lithium was discontinued and replaced with valproic acid.

After the medication change, the manic state resolved, and consciousness and disorientation improved. A few months later, the patient remained stable with no other symptoms except gait instability.

**Conclusions:**

Lithium neurotoxicity is a rare but severe complication of chronic lithium treatment, which can occur even at therapeutic levels (Konieczny K et al. Alpha Psychiatry 2024; 25:190-205). It should be suspected in cases of cerebellar ataxia, parkinsonism, disorientation, or cognitive impairment and requires early intervention to prevent irreversible neurological dysfunction (Schneider MA et al. J Neurosci Nurs. 2019; 51:283-286). In this case, symptoms improved following lithium discontinuation, although sequelae persisted months later, suggesting a potential diagnosis of SILENT syndrome.

We encourage clinicians to consider these symptoms and approach lithium dosing more cautiously in patients undergoing long-term treatment who have maintained psychopathological stability over time (Marmol S et al. Cerebellum 2024; 23:1733-1735).

**Disclosure of Interest:**

None Declared

